# Synergistic Effects of High-Modulus Additives on SBS-Modified Asphalt: Microstructural, Rheological Enhancement, and Dosage-Dependent Performance Optimization

**DOI:** 10.3390/ma18204724

**Published:** 2025-10-15

**Authors:** Qinghua He, Zhuosen Li, Jianqi Huang, Jie Chen, Liujun Zhao, Chengwei Xing, Tong Cui, Jiabiao Zou

**Affiliations:** 1 School of Highway, Chang’an University, Xi’an 710061, Chinaxingcw@chd.edu.cn (C.X.); 2 Guangzhou Expressway Co., Ltd., Guangzhou 510540, China; 3 Poly Changda Engineering Co., Ltd., Guangzhou 511431, China; chenjie891217@163.com (J.C.);

**Keywords:** high-modulus asphalt, SBS-modified asphalt, composite modification, threshold, high-temperature performance

## Abstract

This study systematically investigates the synergistic modification effects of two high-modulus additives on SBS-modified asphalt through microstructural characterization and performance evaluation. Fluorescence microscopic analysis reveals that the additive particles undergo swelling over time and form an interconnected network structure via phase separation dynamics. Rheological tests demonstrate a significant enhancement in high-temperature performance: at the optimal dosage of 10 wt%, the complex modulus increases by approximately 215%, and the rutting factor improves by about 300% compared to the control group. The results from multiple stress creep recovery (MSCR) tests confirm the material’s superior elastic recovery capability and reduced non-recoverable creep compliance. However, the incorporation of the additives adversely affects low-temperature ductility. The penetration of (two distinct high-modulus agents, designated as HMA-A and HMA-B) HMA-B decreases by approximately 36.8% more than that of HMA-A, accompanied by significantly lower low-temperature toughness. A dosage of 10% is identified as the critical threshold, which maximizes rutting resistance while minimizing low-temperature performance degradation. Based on these findings, this paper proposes an integrated design paradigm of “microstructure–performance–dosage,” recommending HMA-B for high-stress pavement channels and HMA-A for regions with substantial temperature variations.

## 1. Introduction

With the vigorous development of Chinese transportation infrastructure, particularly the widespread prevalence of overloaded and heavy-haul traffic, coupled with the increasing frequency of extreme high-temperature weather events during summer attributable to global climate change, asphalt pavements are subjected to increasingly severe challenges during high-temperature seasons [1,2,3,4,5]. Insufficient high-temperature stability has emerged as one of the primary distress mechanisms contributing to the premature deterioration of asphalt pavements, with rutting deformation being particularly prominent and pervasive [6,7,8,9,10,11]. Rutting not only significantly compromises pavement smoothness, riding comfort, and traffic safety but also accelerates moisture-induced damage [12,13,14,15,16]. This substantially reduces the service life of roadways and escalates subsequent maintenance and rehabilitation costs [17,18,19,20,21]. Consequently, enhancing the high-temperature performance of asphalt mixtures, especially that of the asphalt binder, is paramount for ensuring the long-term service performance of pavements and optimizing the return on road infrastructure investment [22,23,24,25].

To address this challenge, polymer-modified asphalt technology has been widely implemented. Among these technologies, Styrene–Butadiene–Styrene (SBS) triblock copolymer-modified asphalt is universally recognized as one of the most prevalent and effective modification approaches currently available [26]. This recognition stems from its superior comprehensive performance, notably its significant enhancement of high-temperature stability, low-temperature crack resistance, and elastic recovery capability [27,28,29]. The SBS modifier effectively enhances the viscoelastic properties of the asphalt and its resistance to permanent deformation through the formation of a three-dimensional network structure within the asphalt matrix [30,31]. This mechanism significantly improves the rut resistance of asphalt mixtures.

However, with the persistent escalation of traffic loads and increasingly severe service environments, conventional Styrene–Butadiene–Styrene (SBS)-modified asphalt may still encounter performance limitations in high-temperature resistance under extreme thermal conditions or heavy-load scenarios [32,33,34]. Against this backdrop, high modulus agents (HMAs) have garnered significant attention in recent years as novel asphalt-modifying components or additives [35]. This interest stems from their demonstrated efficacy in substantially enhancing the complex shear modulus ($G^{*}$), rutting factor ($G^{*}$/sinδ), and softening point of asphalt binders while concurrently reducing their temperature susceptibility [36,37]. These improvements collectively endow asphalt mixtures with superior resistance to permanent deformation [38,39]. Incorporating high-modulus agents presents a critical strategy to substantially enhance the binder’s complex modulus and rutting resistance, thereby addressing these emerging challenges.

The concept of high-modulus asphalt mixtures originates from the intermediate layer materials used in the perpetual pavement designs developed in France and the United States [40,41,42]. The primary objectives for employing high-modulus asphalt mixtures are to enhance pavement rutting resistance, reduce structural layer thicknesses, and improve overall pavement durability [43,44]. High-Modulus Asphalt Concrete (HMAC) emerged in France during the 1980s, where scientists pioneered its development [45]. It was subsequently implemented in practical engineering applications to reduce base course thickness requirements [46]. The French Public Works Research Institute developed these high-modulus mixtures, referred to as Enrobé à Module Élevé (EME), with the objective of improving mechanical properties, including high modulus, good fatigue behavior, and excellent resistance to rutting [47].

Crucially, the development of High-Modulus Asphalt Binders (HMABs) represents the most critical aspect of HMAC research and innovation [48,49]. Furthermore, to date, the incorporation of high-modulus agents into SBS-modified asphalt to prepare composite-modified binders for enhanced performance under more extreme weather events and heavy-duty traffic conditions has not yet been thoroughly investigated. This study focuses on the introduction of high-modulus agents into the well-established SBS-modified asphalt system, exploring a composite modification strategy. The primary objective is to synergistically enhance the high-temperature performance of the SBS-modified asphalt.

By preparing composite-modified asphalt through the blending of HMAs with SBS-modified asphalt, this research systematically investigates the influence of HMA incorporation on the critical high-temperature rheological parameters of the SBS-modified binder. This study aims to elucidate the microscopic state between HMAs and SBS modifiers and evaluate the efficacy of this composite modification technology in overcoming the high-temperature performance limitations of traditional SBS-modified asphalts. The findings are intended to provide novel technical solutions and a theoretical foundation for the development of high-performance, long-life asphalt pavement materials [50,51].

## 2. Materials and Methods

### 2.1. Material

The materials employed in this study comprised SBS-modified asphalt, supplied by Shell Bitumen Xinyue Co., Ltd. (Foshan, China), which served as the base binder, along with two distinct high-modulus agents, designated as HMA-A and HMA-B, procured from China Reagent Network (Shanghai, China). These HMAs are composite modifiers composed of polymers, fibrin, ethylene-vinyl acetate copolymer (EVA), flexible rubber, and functional additives. The physical properties of the HMAs are summarized in Table 1 and Table 2.

Composite-modified asphalt binders were produced by incorporating the aforementioned HMAs into the SBS-modified asphalt base. Key technical properties of the base SBS-modified asphalt are summarized in Table 3.

**Figure 1:**
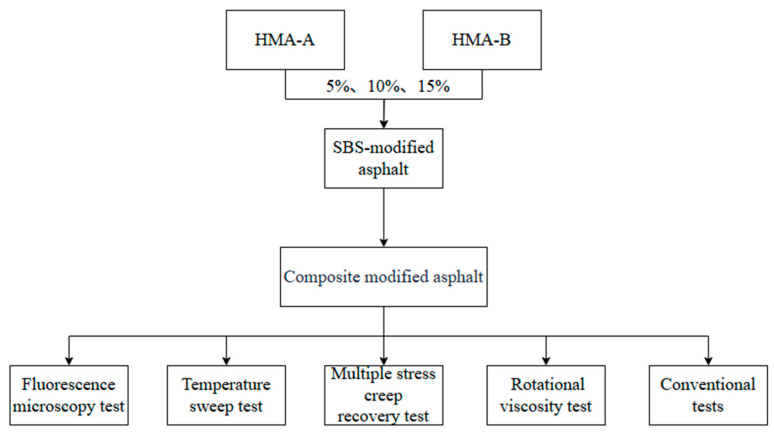
Test flowchart.

The composite-modified asphalt binders were prepared as follows. Each HMA, namely, HMA-A and HMA-B, was incorporated into the base SBS-modified asphalt at three dosage levels, 5%, 10%, and 15%, by the mass of the binder. The blending process was conducted using a high-shear mixer at 175 °C with a rotational speed of 5000 rpm for 60 min. Subsequently, the mixtures were conditioned in a constant-temperature chamber at 170 °C for 2 h to ensure uniformity and stability [34]. The experimental design comprised six modified groups, corresponding to the two HMAs and three incorporation rates, along with a control group consisting of unmodified SBS-modified asphalt (0% HMA). It is noteworthy that the control binder underwent the same thermal and mechanical history as the modified binders to ensure comparability. A schematic diagram of the experimental procedure is provided in Figure 1.

### 2.2. Test Methods

#### 2.2.1. Fluorescence Microscopy Test

Due to the intrinsic property that the saturate and aromatic fractions of asphalt exhibit light-colored phases [56], while the asphaltene and resin fractions appear dark-colored, fluorescence microscopy was employed to investigate the dispersion state of the two HMAs within the SBS-modified asphalt matrix.

The composite-modified asphalt samples prepared with 10% HMA-A and HMA-B and 15% HMA-A and HMA-B, respectively, were examined at room temperature using a fluorescence microscope (Model: XSP-63XA, Xi’an Cewei Photoelectric Technology Co., Ltd., Xi’an, China) at a magnification of 400× [56].

#### 2.2.2. Temperature Sweep (TS) Test

Temperature sweep testing was performed following the AASHTO T 315 protocol on a dynamic shear rheometer (DSR) from TA Instruments to evaluate the viscoelastic properties of the asphalt binders under elevated temperature conditions. Parallel plates with a diameter of 25 mm and a gap setting of 1 mm were employed. Tests were performed under controlled-strain mode (or controlled-stress mode, if specified in the original protocol), maintaining a constant angular frequency of 10 rad/s. The temperature was swept from 46 °C to 82 °C in increments of 6 °C [57]. The resulting key rheological parameters derived from the test included the complex shear modulus ($G^{*}$), the phase angle (δ), and the rutting factor ($G^{*}$/sinδ).

#### 2.2.3. Multiple Stress Creep Recovery (MSCR) Test

Owing to the limited efficacy of the rutting factor (${\;G}^{*}$/sinδ) in evaluating the high-temperature performance of modified asphalt binders, the multiple stress creep recovery test was performed in accordance with ASTM D7405 [58] to evaluate the creep and recovery properties of the asphalt binder. Tests were conducted at 64 °C using parallel-plate geometry (25 mm diameter, 1 mm gap) on a dynamic shear rheometer. The standard MSCR protocol comprised 20 creep recovery cycles at a shear stress of 0.1 kPa and 10 creep recovery cycles at a shear stress of 3.2 kPa. Each cycle consisted of a 1 s creep phase under constant shear stress and a 9 s recovery phase under nominally zero stress [59].

#### 2.2.4. Rotational Viscosity (RV) Test

The rotational viscosity test, conducted in accordance with ASTM D4402 [60], was used to characterize the rheological behavior of construction-grade asphalt binders. Utilizing a Brookfield rotational viscometer equipped with an SC4-27 spindle rotating at 20 rpm, apparent viscosity values were quantified at discrete temperatures of 145 °C, 155 °C, 165 °C, and 175 °C. This methodology provides critical data for determining optimal mixing and compaction temperatures and evaluating temperature susceptibility during placement operations.

#### 2.2.5. Conventional Tests

Conventional tests were performed in compliance with the specification JTGE 20-2011.

Ductility testing at 5 °C quantified plastic deformation resistance through elongation measurement, providing an empirical indicator of pavement cracking resistance under thermal stress.

Softening point determination employed a glycerin bath with controlled heating at 5 ± 0.5 °C/min, establishing the temperature at which bitumen reaches specified viscosity (equivalent to 1200 Poise), thereby reflecting temperature susceptibility and high-temperature stability.

Penetration testing at 25 °C measured the depth (in 0.1 mm units) that a standardized needle achieves under 100 g/5 s loading, serving as an empirical viscosity index that correlates with shear resistance and consistency grading.

## 3. Results

### 3.1. Fluorescence Microscopy Test

**Figure 2:**
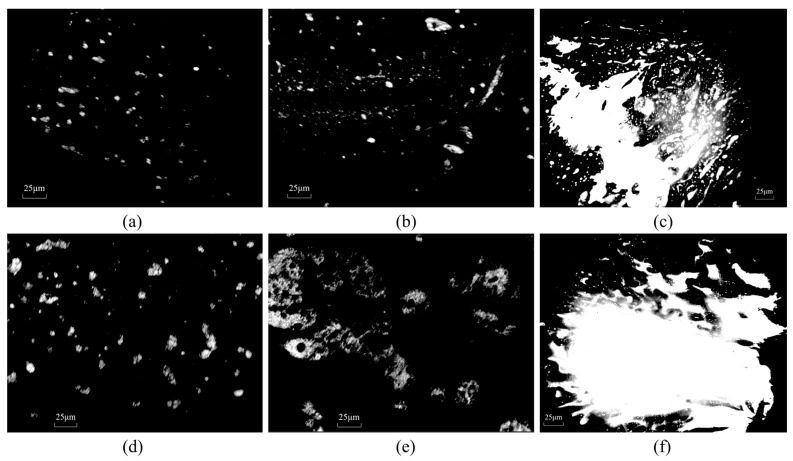
Fluorescence micrographs: (**a**) 10% HMA-A composite-modified asphalt at 30 min; (**b**) 10% HMA-A composite-modified asphalt at 60 min; (**c**) 15% HMA-A composite-modified asphalt at 60 min; (**d**) 10% HMA-B composite-modified asphalt at 30 min; (**e**) 10% HMA-B composite-modified asphalt at 60 min; (**f**) 15% HMA-B composite-modified asphalt at 60 min.

Figure 2 depicts the morphological development of the composite-modified asphalt binders as captured by fluorescence microscopy after 30 and 60 min of mixing. At 30 min intervals, the polymer was observed to be a droplet-like dispersion within the 10% HMA-A and HMA-B asphalt matrix and initially formed discontinuities. After 60 min of mixing, the polymer particle size increases significantly. Following 60 min of mixing, an increase in polymer particle size becomes evident. This morphological change is attributed to enhanced polymer swelling dynamics, driven by the rapid migration of maltene compounds coupled with the gradual expansion of the polymer-rich phase [61]. Furthermore, the modifier droplets exhibit a propensity to form interconnected networks, likely resulting from particle swelling and subsequent coalescence [56]. As shown in Figure 2c,f, the modifier can no longer be evenly dispersed in the asphalt when the HMA content is 15%. Instead, particles tend to aggregate to form large-scale domains rather than continuous networks.

From a morphological perspective, discernible differences were observed between the HMA-A and HMA-B composite-modified asphalt binders in terms of dispersion state and network-forming capability. Specifically, the particle diameter within the HMA-A-modified system was smaller than that in the HMA-B-modified binder. Furthermore, after 60 min of mixing, the area occupied by the network-like structure formed in the HMA-A-modified asphalt was comparatively smaller. These morphological distinctions may be attributed to the relatively lower polymer content in HMA-A, which also accounts for its lower density compared to HMA-B and the correspondingly lower softening point of the HMA-A composite-modified asphalt at equivalent dosage levels. This microstructural evidence substantiates that the more pronounced enhancement in high-temperature performance imparted by HMA-B is fundamentally due to its higher polymer content.

### 3.2. Temperature Sweep Test

**Figure 3:**
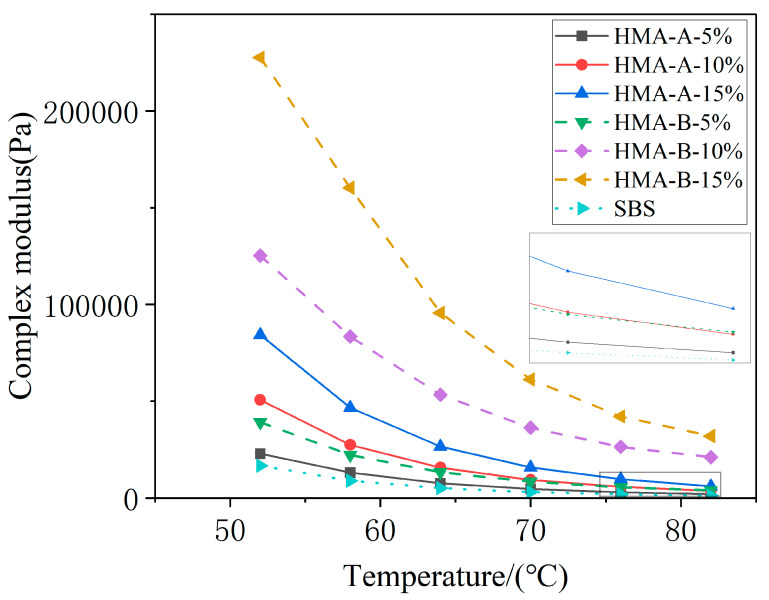
Complex modulus of base-modified SBS asphalt and HMA-A- and HMA-B-modified composites.

The complex modulus ($G^{*}$) of asphalt quantifies its total resistance to repeated shear deformation, equivalent to the stiffness modulus. As illustrated in Figure 3, $G^{*}$ decreases progressively with increasing temperature. Elevated temperatures intensify molecular thermal motion, enhancing chain segment mobility and consequently increasing material deformability under equivalent stress. This results in a reduction in the stiffness modulus, consistent with the observed decline in ${\;G}^{*}$ at higher temperatures. At identical temperatures, however, the incorporation of a high-modulus additive substantially enhances ${\;G}^{*}$. This enhancement primarily arises from the formation of particulate and network-like microstructures within the asphalt matrix upon blending with the additive. Fluorescence microscopy confirms the presence of these granular and interconnected network structures. Under external loading, these features act as stress concentration sites, absorbing excess energy. Consequently, higher stress levels are required to achieve equivalent deformation, leading to significantly elevated $\;G^{*}$ values for the composite-modified asphalt compared to SBS-modified asphalt at the same temperature. Comparative analysis further reveals a pronounced improvement in ${\;G}^{*}$ attributable to the additive, a trend corroborated by the evolution of the rutting factor.

**Figure 4:**
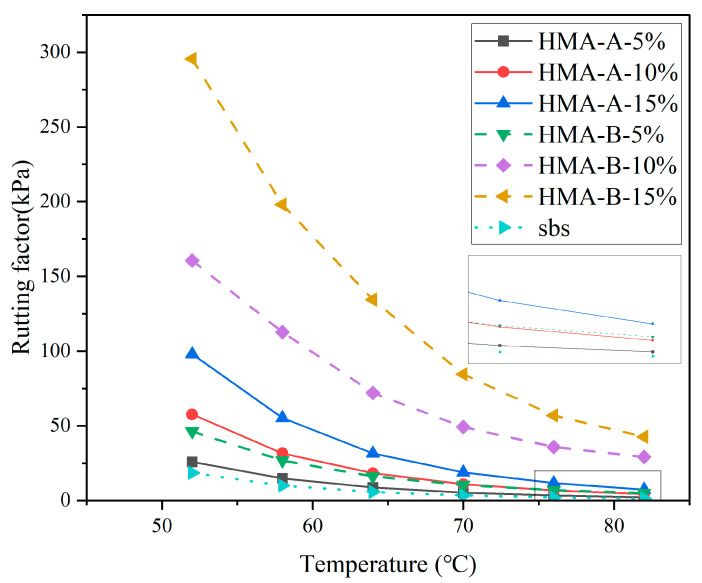
Rutting factor of base-modified SBS asphalt and HMA-A- and HMA-B-modified composites.

The rutting factor ($G^{*}$/sinδ) serves as an indicator of the asphalt’s high-temperature resistance to shear deformation, with higher values signifying superior rutting resistance. Figure 4 demonstrates that the high-modulus composite-modified asphalt exhibits a greater rutting factor than SBS-modified asphalt. This indicates that the high-modulus additive effectively enhances the asphalt’s high-temperature shear resistance. Furthermore, this resistance increases proportionally to the additive dosage.

### 3.3. Multiple Stress Creep Recovery (MSCR) Test

**Figure 5:**
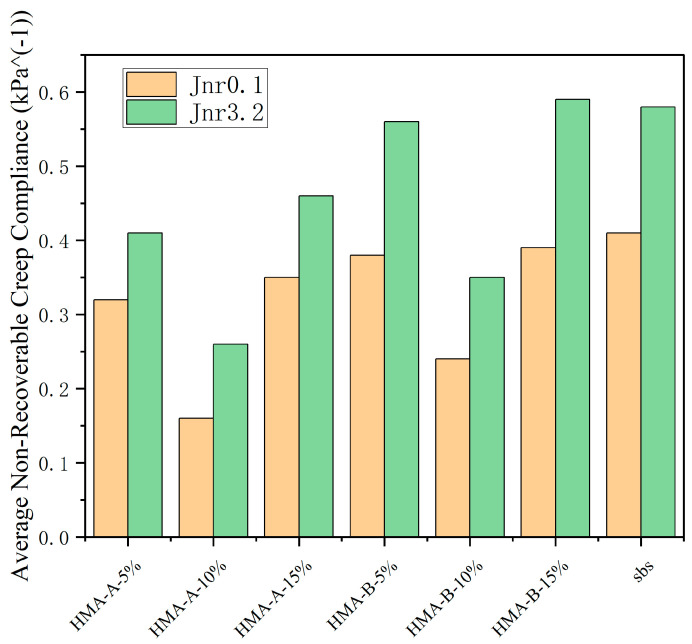
Average non-recoverable creep compliance of base-modified SBS asphalt and HMA-A- and HMA-B-modified composites.

**Figure 6:**
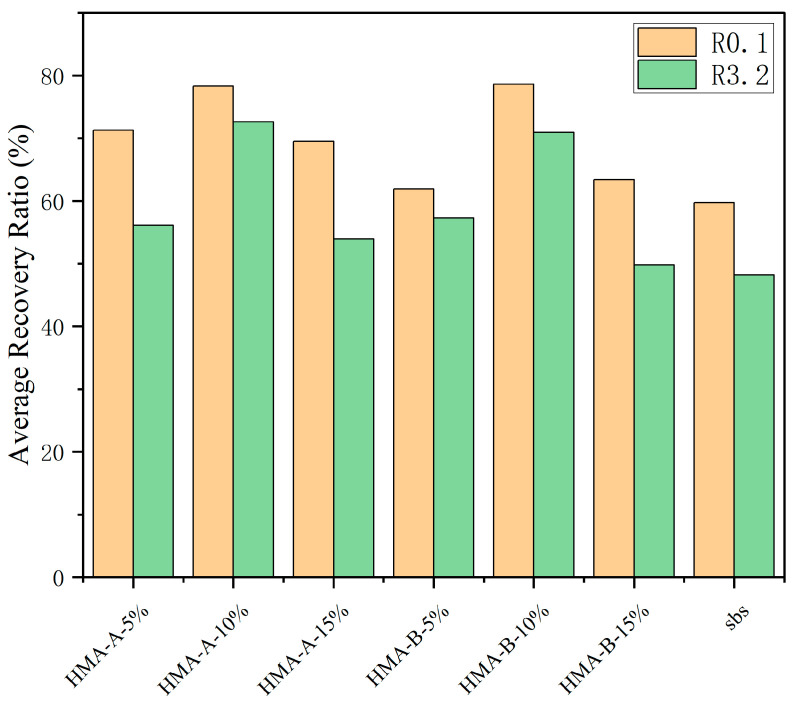
Average recovery ratio of base-modified SBS asphalt and HMA-A- and HMA-B-modified composites.

The MSCR test provides critical insights into the high-temperature performance and elastic recovery capabilities of the modified binders under sustained stress. As illustrated in Figure 5 and Figure 6, the incorporation of high-modulus agents into SBS-modified asphalt significantly improved its resistance to permanent deformation. Both HMA-A and HMA-B enhanced the binder’s performance, as evidenced by the following key trends:

1. Increased Average Recovery (R%). The addition of HMAs resulted in a substantial increase in the average percent recovery compared to the control SBS-modified asphalt (Figure 6). This indicates an enhancement in the elastic response and stress recovery capacity of the composite binders.
2. Reduced Non-Recoverable Creep Compliance. Concurrently, the average non-recoverable creep decreased markedly upon HMA modification (Figure 5). Lower Jnr values signify a greater resistance to permanent, viscous deformation under load.
3. Optimum Dosage at 10%. For both HMA-A and HMA-B, the optimal improvement in MSCR parameters manifested as the highest R%.
4. Performance Decline Beyond Optimum Dosage. Notably, when the HMA dosage exceeded 10%, a subsequent decline in performance was observed for both modifiers. The average percent recovery (R%) decreased, and the non-recoverable creep increased compared to their respective values at the 10% dosage level.
5. These MSCR results collectively demonstrate that the HMAs effectively enhance the high-temperature elastic recovery and reduce the permanent deformation susceptibility of SBS-modified asphalt. The existence of a clear performance optimum at 10% dosage suggests potential saturation or detrimental microstructural changes occurring at higher additive concentrations.
6. A performance reversal was observed when the HMA content exceeded 10%. This phenomenon may be attributed to the limited compatibility between the asphalt matrix and the modifier. Beyond this critical dosage, excessive modifiers could no longer be uniformly dispersed within the asphalt. Instead, the particles tended to agglomerate, forming large-scale domains rather than a continuous network. These macroscopic, rigid aggregates create weak interfacial zones with the surrounding softer asphalt matrix. Under mechanical loading, stress concentrations readily develop at these interfaces, leading to interfacial debonding or microcrack initiation. Once interfacial failure occurs, these aggregates act as internal defects, thereby accelerating material damage and promoting irreversible deformation. The elevated non-recoverable creep observed in SBS-modified asphalt may also be attributed to this mechanism.

### 3.4. Rotational Viscosity (RV) Test

**Figure 7:**
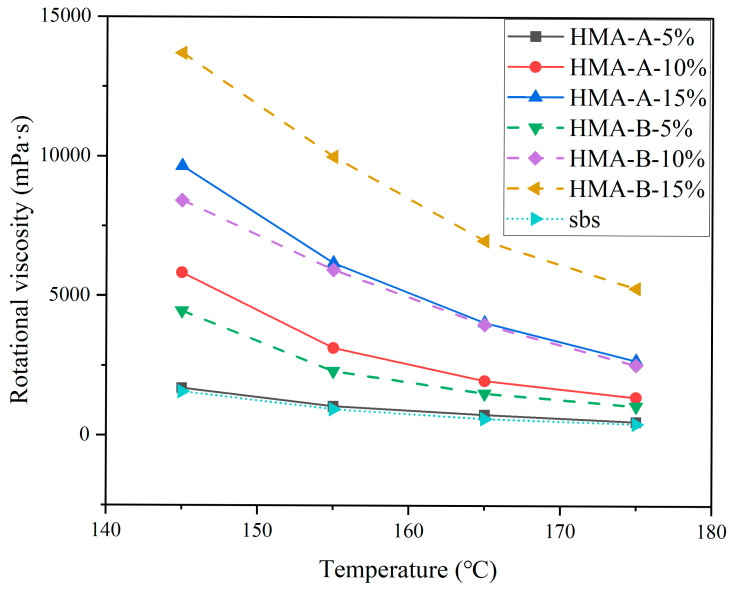
Rotational viscosity of base-modified SBS asphalt and HMA-A- and HMA-B-modified composites.

As indicated by the test results presented in Figure 7, the viscosity of asphalt increases proportionally with the dosage of both high-modulus additives. This demonstrates that incorporating these additives enhances the viscous properties of asphalt. Notably, at equivalent dosage levels, HMA-B exhibits a more pronounced effect on viscosity enhancement compared to HMA-A. Furthermore, the viscosity of all asphalt samples decreases with rising temperature, attributable to the gradual transition of asphalt from a viscoelastic solid state to a flowable liquid state, consequently reducing its internal resistance to deformation. Although the rotational viscosity of the composite-modified asphalt significantly increases with higher HMA dosage—particularly at elevated concentrations and in HMA-B-modified systems—its practical constructability remains largely unaffected. Rheological measurements confirm that all samples retain a workable state suitable for pumping, mixing, and paving within standard construction temperature ranges. The marked reduction in viscosity with rising temperature indicates that moderately increasing the construction temperature can effectively modulate the binder’s workability, ensuring homogeneous coating and compaction quality of the mixture. Moreover, the enhanced viscosity imparted by the high-modulus additives represents an acceptable techno-economic trade-off, given their substantial improvement in high-temperature deformation resistance.

### 3.5. Conventional Tests

**Figure 8:**
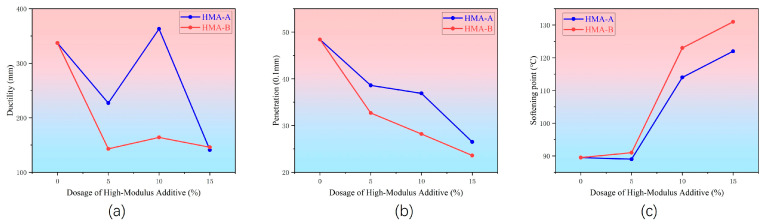
Results of conventional tests: (**a**) ductility, (**b**) penetration, (**c**) softening point.

The ductility test results, as illustrated in Figure 8a, demonstrate that the incorporation of high-modulus agents exerts a significant influence on the low-temperature ductility of SBS-modified asphalt, following a non-monotonic trend. Specifically, when the dosage increases to 10%, the ductility value reaches its peak, indicating that at this critical dosage, the composite material achieves an optimal enhancement in high-temperature performance while retaining a certain degree of low-temperature deformability. However, as the dosage is further increased to 15%, the ductility of both modified asphalts declines markedly, falling below that of the baseline SBS asphalt. This suggests that an excessively high content of high-modulus agent severely compromises the low-temperature crack resistance of the asphalt. A comparison between the two high-modulus agents reveals that the adverse effect of HMA-B on low-temperature ductility is consistently more pronounced than that of HMA-A. This phenomenon correlates with fluorescence microscopy observations. Due to its higher polymer content, HMA-B forms a more developed and rigid three-dimensional network within the asphalt. While this structure considerably enhances the high-temperature stiffness of the material, it simultaneously restricts its plastic deformation capacity at low temperatures, resulting in reduced toughness. Consequently, although HMA-B demonstrates superior performance in enhancing high-temperature properties, it also entails a more significant sacrifice in low-temperature performance.

Penetration test results documented in Figure 8b confirm compliance of SBS-modified asphalt with technical specifications (T/CECS G:K44-02-2020) [62]. Upon introducing high-modulus additives, the following occurs:

HMA-A induces a monotonous penetration response, characterized by gradual attenuation with increasing dose. HMA-B shows the same trend.

Critically, HMA-B achieves about 36.8% greater penetration reduction than HMA-A at equivalent concentrations. This unequivocally establishes HMA-B’s superior efficacy in enhancing asphalt consistency and rigidity relative to HMA-A.

The data obtained from the softening point test are documented in Figure 8c. Analysis of the data reveals that the softening point of asphalt exhibits a continuous increase with the addition of the two types of high-modulus agents, maintaining a direct proportionality with the dosage of the high-modulus agents. Moreover, under the same dosage conditions, HMA-B demonstrates a more pronounced enhancement effect compared to HMA-A.

## 4. Discussion

This study systematically investigated the composite modification effects of two types of high-modulus additives on SBS-modified asphalt through fluorescence microscopy, rheological characterization, and conventional performance tests. The main conclusions are as follows.

1. Microstructural

Fluorescence micrographs revealed that the high-modulus additives gradually underwent particle swelling and formed network structures within the asphalt matrix over time. This process is primarily driven by phase separation dynamics, whereby the additive particles coalesce to form continuous or semi-continuous networks, thereby enhancing the overall stiffness and deformation resistance of the asphalt. Owing to its higher polymer content, HMA-B developed a more pronounced network structure, which accounts for its superior high-temperature performance. However, when the content of HMAs is too high, the modifier can no longer disperse uniformly in the asphalt. Instead, particles tend to aggregate to form large-scale domains rather than continuous networks.

2. Performance Enhancement and Trade-offs

The incorporation of high-modulus additives significantly improved the high-temperature performance (e.g., complex modulus, rutting factor), elastic recovery, and viscosity of the SBS-modified asphalt. However, it also adversely affected the low-temperature ductility and toughness. Notably, at dosages exceeding 10%, a marked decline in low-temperature performance was observed, indicating the necessity of carefully controlling additive content when optimizing high-temperature properties.

3. Existence of an Optimal Dosage Threshold

The study identified 10% as the optimal dosage for the high-modulus additives, at which the enhancement in high-temperature performance was most significant, while the reduction in low-temperature performance was minimized. This dosage corresponds to the critical point at which an additive network begins to form without excessive agglomeration. Beyond this threshold, reduced dispersibility and particle aggregation led to a decline in performance.

4. Differential Effects of Additive Type

HMA-B outperformed HMA-A in enhancing high-temperature stiffness, viscosity, and rutting resistance. However, it also exerted a more detrimental effect on low-temperature performance, particularly in terms of ductility. These findings suggest that HMA-B is more suitable for high-temperature and heavy-load road sections, whereas HMA-A is better suited for regions with significant temperature variations. Selection should be based on specific engineering conditions.

5. Engineering Application Recommendations

This research provides a theoretical framework linking “Microstructural –performance–dosage” for the design of high-modulus composite-modified asphalt. It is recommended that the type and dosage of additives be selected according to climatic conditions and traffic loads to achieve an optimal balance between high- and low-temperature performance.

6. Future Research

While this study has focused on the rheological and fundamental properties of the composite-modified asphalt binders, future work will investigate the performance of corresponding asphalt mixtures to further validate the practical applicability of these materials.

This study validated the efficacy of two high-modulus agents and innovatively incorporated them into SBS-modified asphalt to investigate the pertinent properties of the resulting composite-modified binder, thereby addressing the challenges posed by prospective extreme high-temperature events and heavy-duty traffic conditions.

## Figures and Tables

**Table 1:** The fundamental physical characteristics of HMA-A.

The Fundamental Physical Characteristics	Result	Methods
Outlook	Black particles	Estimate visually
Softening point/(°C)	≥160	GB/T 2294-2019 [52]
Density/(kg/$m^{3}$)	952	Density bottle method
Particle diameter/(mm)	≤5.0	Sieve mesh
Polymer content/(%)	≥90%	GB/T 27761-2011 [53]
Melt index/(g/10 min)	5–7	GB/T 3682.1-2018 [54]

**Table 2:** The fundamental physical characteristics of HMA-B.

The Fundamental Physical Characteristics	Result	Methods
Outlook	Black particles	Estimate visually
Softening point/(°C)	≥160	GB/T 2294-2019
Density/(kg/$m^{3}$)	1198	Density bottle method
Particle diameter/(mm)	≤5.0	Sieve mesh
Polymer content/(%)	≥95%	GB/T 27761-2011
Melt index/(g/10 min)	7–10	GB/T 3682.1-2018

**Table 3:** Main technical indicators of SBS asphalt.

Detection Parameters	Technical Requirements	Test Results	Test Methods
Penetration (25 °C, 100 g, 5 s)/(0.1) mm	40~60	48.4	JTGE 20-2011 [55]
Penetration Index (PI)	≥0	2.08	JTGE 20-2011
Ductility (5 °C, 5 cm/min)/(cm)	≥20	33.7	JTGE 20-2011
Softening point (TR&B)/(°C)	≥60	89.5	JTGE 20-2011
Flash point/(°C)	≥230	321	JTGE 20-2011

## Data Availability

The original contributions presented in this study are included in the article. Further inquiries can be directed to the author.

## References

[1] Wang L., Wei J.C., Wu W.J., Zhang X.M., Xu X.Z., Yan X.P. (2022). Technical development and long-term performance observations of long-life asphalt pavement: A case study of Shandong Province. J. Road Eng..

[2] Li Q., Yang H., Ni F., Ma X., Luo L. (2015). Cause analysis on permanent deformation for asphalt pavements using field cores. Constr. Build. Mater..

[3] Hao Y., Ye Y., Zhuang C., Hou F. (2022). Research on High-Temperature Evaluation Indexes and Performance of Qingchuan Rock-SBS Composite Modified Asphalt. Materials.

[4] Xing C., Tang S., Chang Z., Han Z., Li H., Zhu B. (2024). A comprehensive review on the plant-mixed cold recycling technology of emulsified asphalt: Raw materials and factors affecting performances. Constr. Build. Mater..

[5] Tian T., Jiang Y., Yi Y., Nie C. (2025). The splitting fatigue properties of ultra-large particle size asphalt mixture under the coupling effect of temperature and load. Eng. Fract. Mech..

[6] Mu Y., Fu Z., Liu J., Li C., Dong W., Dai J. (2020). Evaluation of High-Temperature Performance of Asphalt Mixtures Based on Climatic Conditions. Coatings.

[7] Xu S., Ruan P., Lu Z., Liang L., Han B., Hong B. (2021). Effects of the high temperature and heavy load on the rutting resistance of cold-mix emulsified asphalt mixture. Constr. Build. Mater..

[8] Zhang K., Wang S., Yang W., Zhong X., Liang S., Tang Z., Quan W. (2023). Influence of temperature and humidity coupling on rutting deformation of asphalt pavement. Sci. Eng. Compos. Mater..

[9] Shang K., Wan C., Guo M., Zhou C., Jiang Y., Ren J. (2025). Temperature-Dependent Model of Rutting Behavior for Connected Layer Mixtures in Flexible Base Asphalt Pavement. Materials.

[10] Zhang Q.-s., Chen Y.-l., Li X.-l. (2012). Rutting in Asphalt Pavement under Heavy Load and High Temperature. Asphalt Material Characterization, Accelerated Testing, and Highway Management, Proceedings of the GeoHunan International Conference, Changsha, China, 3–6 August 2009.

[11] Li N., Zhan H., Yu X., Tang W., Yu H., Dong F. (2021). Research on the high temperature performance of asphalt pavement based on field cores with different rutting development levels. Mater. Struct..

[12] Fares A., Wong M.-N., Zayed T., Faris N. (2025). Impact of Rutting on Traffic Safety: A Synthesis of Research Findings. Appl. Sci..

[13] Fares A., Zayed T., Abdelkhalek S., Faris N., Muddassir M. (2024). Rutting measurement in asphalt pavements. Autom. Constr..

[14] Rahman M.A., Ghabchi R., Zaman M., Ali S.A. (2021). Rutting and moisture-induced damage potential of foamed warm mix asphalt (WMA) containing RAP. Innov. Infrastruct. Solut..

[15] Luo L., Yang S.-H., Oeser M., Liu P. (2024). Moisture damage mechanism of asphalt mixtures containing reclaimed asphalt pavement binder: A novel molecular dynamics study. J. Clean. Prod..

[16] Neupane P., Wu S. (2025). A comprehensive review of moisture damage in asphalt mixtures: Mechanisms, evaluation methods, and mitigation strategies. Constr. Build. Mater..

[17] Figliozzi M., Johnson P., Monsere C., Nordback K. (2014). Methodology to Characterize Ideal Short-Term Counting Conditions and Improve AADT Estimation Accuracy Using a Regression-Based Correcting Function. J. Transp. Eng..

[18] Lee J., Guan H., Loo Y.-C., Blumenstein M. (2014). Development of a Long-Term Bridge Element Performance Model Using Elman Neural Networks. J. Infrastruct. Syst..

[19] Lu Q., Sha A., Jiang W., Jiao W., Chen Y., Feng Z., Wang S., Li Z. (2025). Cooling water-retentive semi-flexible pavement with light-colored grout and recycled materials. Constr. Build. Mater..

[20] Li X., Sha A., Jiao W., Cao Y., Song R. (2025). Strain response and creep behavior of asphalt mixture based on multi-damage fractional visco-elasto-plastic constitutive model. Constr. Build. Mater..

[21] Fu Z., Song R.M., Qin W., Shi K., Ma F., Li J.R., Li C. (2025). Investigation on the low temperature rheological properties of polymer modified asphalt. J. Traffic Transp. Eng.-Engl. Ed..

[22] Chen Y.J., Sha A.M., Jiang W., Lu Q., Du P.D., Hu K., Li C. (2025). Eco-friendly bismuth vanadate/iron oxide yellow composite heat-reflective coating for sustainable pavement: Urban heat island mitigation. Constr. Build. Mater..

[23] Chen Y., Sha A., Cao Y., Lu Q., Li C., Hu K., Li Z. (2025). Iron-chromium based high infrared reflectance coating for cooling asphalt pavements towards low-carbon cities. Constr. Build. Mater..

[24] Zhang Y.J., Sha A.M., Jiao W.X., Shi K., Ren X.Y., Du P.D., Li X.Z. (2025). Investigation on the self-healing performance of asphalt binder under the coupling effect of multiple factors. Constr. Build. Mater..

[25] Zhang F., Zhu J.Q., Sun Y.X., Benny C.M., Wang D., Falchetto A.C. (2025). Effect analysis of using tall oil pitch (TOP) to partially extend bitumen in asphalt pavements: Comparison of different TOPs. Road Mater. Pavement Des..

[26] Raeis-Hosseini N., Rho J. (2017). Metasurfaces Based on Phase-Change Material as a Reconfigurable Platform for Multifunctional Devices. Materials.

[27] Liu J., Bai Y.X., Song Z.Z., Kanungo D.P., Wang Y., Bu F., Chen Z.H., Shi X. (2020). Stabilization of sand using different types of short fibers and organic polymer. Constr. Build. Mater..

[28] Baldovino J.D.A., Izzo R.L.D., da Silva E.R., Rose J.L. (2020). Sustainable Use of Recycled-Glass Powder in Soil Stabilization. J. Mater. Civ. Eng..

[29] Fu Y.X., Soldera M., Wang W., Voisiat B., Lasagni A.F. (2019). Picosecond Laser Interference Patterning of Periodical Micro-Architectures on Metallic Molds for Hot Embossing. Materials.

[30] Qi X.Q., Zhang S.L., Wang T.T., Guo S.Y., Ren R. (2021). Effect of High-Dispersible Graphene on the Strength and Durability of Cement Mortars. Materials.

[31] Li Z., Zhang J.Y., Wang R., Monti G., Xiao Y. (2020). Design Embedment Strength of Plybamboo Panels Used for GluBam. J. Mater. Civ. Eng..

[32] Xie X., Zhang Y., Li G., Liu C., SiMa X., Liu C., Si B., He Y., Shao J. (2024). Study on the composition and properties of EME-SBS-Nano ZnO high modulus asphalt material. Sci. Rep..

[33] Khan I., Bilal M., Khaliq W., Khan N., Khahro S.H., Memon Z.A., Malik M.A. (2024). Evaluating the dynamic response and phase angle behavior of SBS-modified asphalt mixtures for enhanced pavement performance. Sci. Rep..

[34] Li H., Xing C., Zhu B., Zhang X., Gao Y., Tang S., Cheng H. (2025). Comparative analysis of four styrene-butadiene-styrene (SBS) structure repair agents in the rejuvenation of aged SBS-modified bitumen. Constr. Build. Mater..

[35] Xu X., Lu G., Yang J., Liu X. (2020). Mechanism and Rheological Properties of High-Modulus Asphalt. Adv. Mater. Sci. Eng..

[36] Wang Y., Guo S., Pei Z., Zhan S., Lin S., Ma K., Lei J., Yi J. (2024). Study of the Properties and Modification Mechanism of SBS-Modified Asphalt by Dry Process. Materials.

[37] Wang L., Liang F., Li Z., Zhao Q. (2023). Investigation on the Rheological Properties and Microscopic Characteristics of Graphene and SBR Composite Modified Asphalt. Coatings.

[38] Han D., Hu G., Zhang J. (2023). Study on Anti-Aging Performance Enhancement of Polymer Modified Asphalt with High Linear SBS Content. Polymers.

[39] Yan C., Huang W., Ma J., Xu J., Lv Q., Lin P. (2020). Characterizing the SBS polymer degradation within high content polymer modified asphalt using ATR-FTIR. Constr. Build. Mater..

[40] Kamran F., Ghasemirad A., Moghaddam T.B., Bayat A., Hashemian L. (2022). Performance Evaluation of High Modulus Asphalt Concrete (HMAC) Prepared Using Asphaltenes-Modified Binders. J. Test. Eval..

[41] Zhao J., Gao Z.W., Wu W.J., Tian Y., Fan J.T., Wang X.C. (2025). Development of a novel cement-based microwave deicing functional overlay using waterborne epoxy resin: Heating properties, influencing factors, and mechanism. Constr. Build. Mater..

[42] Shi K., Ma F., Falchetto A.C., Fu Z., Yuan D.D., Song R.M., Dai J.S., Wang H.P. (2025). Comprehensive review on the composition, influence, and inhibition of asphalt fumes. J. Traffic Transp. Eng.-Engl. Ed..

[43] Lee H.J., Lee J.H., Park H.M. (2007). Performance evaluation of high modulus asphalt mixtures for long life asphalt pavements. Constr. Build. Mater..

[44] Han Z.Q., Tang J.Q., Hu L.Q., Jiang W., Sha A.M. (2025). Automated measurement of asphalt pavement rut depth using smartphone imaging. Autom. Constr..

[45] Wang D., Falchetto A.C., Riccardi C., Poulikakos L., Hofko B., Porot L., Wistuba M.P., Baaj H., Mikhailenko P., Moon K.H. (2019). Investigation on the combined effect of aging temperatures and cooling medium on rheological properties of asphalt binder based on DSR and BBR. Road Mater. Pavement Des..

[46] Zaumanis M., Arraigada M., Poulikakos L.D. (2020). 100% recycled high-modulus asphalt concrete mixture design and validation using vehicle simulator. Constr. Build. Mater..

[47] Ullah A., Wen H.P., Ullah Z., Ali B., Khan D. (2024). Evaluation of high modulus asphalts in China, France, and USA for durable road infrastructure, a theoretical approach. Constr. Build. Mater..

[48] Zhang J., Walubita L.F., Faruk A.N.M., Karki P., Simate G.S. (2015). Use of the MSCR test to characterize the asphalt binder properties relative to HMA rutting performance—A laboratory study. Constr. Build. Mater..

[49] Chaturabong P., Bahia H.U. (2018). Effect of moisture on the cohesion of asphalt mastics and bonding with surface of aggregates. Road Mater. Pavement Des..

[50] Li Z., Yu X., Liang Y., Wu S. (2021). Carbon Nanomaterials for Enhancing the Thermal, Physical and Rheological Properties of Asphalt Binders. Materials.

[51] Xue Z., Xu W. (2023). A Study on High and Low Temperature Rheological Properties and Oil Corrosion Resistance of Epoxy Resin/SBS Composite Modified Bitumen. Polymers.

[52] (2019). Determination of Softening Point in Solid Products of Coal Carbonization.

[53] (2011). Standard Test Method of Mass Loss and Residue Measurement Validation of Thermogravimetric Analyzers.

[54] (2018). Plastics—Determination of the Melt Mass-Flow Rate (MFR) and Melt Volume-Flow Rate (MVR) of Thermoplastics—Part 1: Standard Method.

[55] (2011). Standard Test Methods of Bitumen and Bituminous Mixtures for Highway Engineering.

[56] Zou X., Sha A., Jiang W., Huang X. (2015). Modification mechanism of high modulus asphalt binders and mixtures performance evaluation. Constr. Build. Mater..

[57] Lu Q., Sha A., Jiao W., Shi K., Peng Z., Song R. (2024). Synergistic effects of sulfur and polyphosphoric acid additives in warm mix bio-rejuvenated asphalt modification. Constr. Build. Mater..

[58] (2024). Standard Test Method for Multiple Stress Creep and Recovery (MSCR) of Asphalt Binder Using a Dynamic Shear Rheometer.

[59] Lu Q., Sha A., Jiao W., Shi K., Li Z., Chen Y., Du P., Peng Z., Song R. (2024). Waste coffee biochar and bi-oil composite modified rejuvenated asphalt: Preparation, characterization, and performance evaluation. Constr. Build. Mater..

[60] (2002). Standard Test Method for Viscosity Determination of Asphalt at Elevated Temperatures Using a Rotational Viscometer.

[61] Polacco G., Stastna J., Biondi D., Zanzotto L. (2006). Relation between polymer architecture and nonlinear viscoelastic behavior of modified asphalts. Curr. Opin. Colloid Interface Sci..

[62] (2020). Technical Specification of SBS and Crumb Rubber Modified Asphalt Mixture for Highway Pavement.

